# Akiferidin suppresses cervical cancer growth and angiogenesis via the VEGF/DLL4-Notch pathway

**DOI:** 10.3389/fphar.2026.1803135

**Published:** 2026-04-21

**Authors:** Jingyuan Lan, Jun Lu, Tuerhong Dina, Yuxuan Li, Dexi Wang, Haiying Zhang

**Affiliations:** 1 Department of Obstetrics and Gynecology, Zhejiang Provincial Clinical Research Center for Gynecological Diseases, The Second Affiliated Hospital of Wenzhou Medical University, Wenzhou, China; 2 Department of Pharmacy, Affiliated Hospital of Traditional Chinese Medicine of Xinjiang Medical University, Urumqi, China; 3 Xinjiang Key Laboratory of Processing and Research of Traditional Chinese Medicine, Urumqi, China; 4 Office of Drug Clinical Trial Institution, Affiliated Hospital of Traditional Chinese Medicine of Xinjiang Medical University, Urumqi, China

**Keywords:** akiferidin, angiogenesis, cervical cancer, Ferula songorica, VEGF/DLL4-notch pathway

## Abstract

Cervical cancer remains a major gynecological malignancy with a substantial global burden, particularly in low- and middle-income regions. Tumor angiogenesis driven by the VEGF/DLL4–Notch axis promotes cervical cancer progression, and the anti-VEGF antibody bevacizumab shows limited clinical benefit because of acquired resistance. Our prior work identified anti-tumor activity in the Xinjiang-native medicinal plant *Ferula songorica* Pall. ex Spreng, but the effects and mechanisms of its principal bioactive metabolite, akiferidin, in cervical cancer have not been defined. Here, we evaluated akiferidin’s anti-tumor and anti-angiogenic activities in cervical cancer, examined its combinatorial potential with bevacizumab, and explored the underlying mechanisms. We developed and validated an HPLC method to quantify akiferidin in *F. songorica* ethanol extract. *In vitro*, we used MTT, wound-healing, Transwell, and Matrigel tube-formation assays to assess akiferidin’s effects on U14 cell proliferation, migration, invasion, and HUVEC angiogenesis. *In vivo*, we evaluated the efficacy and safety of akiferidin alone and in combination with bevacizumab in U14 xenograft-bearing C57BL/6 mice. Modulation of the VEGF/DLL4–Notch pathway was examined by IHC and Western blot, and direct binding between akiferidin and VEGF-A was investigated by molecular docking followed by 100 ns molecular dynamics simulation. The HPLC method demonstrated excellent robustness (*R* ^2^ = 0.999, all RSD<2%), yielding an akiferidin content of 57.45 ± 1.42 mg/g in the extract. *In vitro*, akiferidin inhibited U14 cell proliferation, migration, invasion, and HUVEC tube formation in a concentration-dependent manner (IC_50_ = 8.06 μg/mL). *In vivo*, akiferidin suppressed tumor growth in a dose-dependent fashion without overt systemic toxicity, and its combination with bevacizumab markedly enhanced antitumor efficacy while reducing tumor microvessel density. Mechanistically, akiferidin significantly downregulated key proteins in the VEGF/DLL4-Notch pathway, with greater inhibition observed in the combination group. Molecular docking and dynamics confirmed stable binding between akiferidin and VEGF-A at five key sites. In conclusion, akiferidin exerts potent anti–cervical cancer activity by inhibiting angiogenesis through modulation of the VEGF/DLL4-Notch pathway, and its combination with bevacizumab enhances antitumor efficacy with favorable *in vivo* safety, representing a promising therapeutic strategy for cervical cancer.

## 1 Introduction

Cervical cancer is a common malignant tumor that seriously threatens the health of women and is also a key focus of global public health. In 2022, there were 661,000 new cases of cervical cancer worldwide, with 348,000 deaths, mainly caused by infection with high-risk human papillomavirus (HPV). It brings a heavy burden to patients, families and society, and the harm is particularly prominent in low- and middle-income areas (Zhou et al., 2025). From 1990 to 2021, the number of new cases of cervical cancer, deaths, and disability-adjusted life years (DALYs) worldwide increased by 62.9%, 40.2%, and 33.6% respectively. However, the age-standardized incidence rate, mortality rate and DALYs rate all showed a downward trend, with differences existing in regions with different socio-demographic index (SDI). The disease burden in low-SDI areas is still relatively high. Unsafe sexual behavior is the primary risk factor for cervical cancer DALYs, while smoking is a secondary risk factor (Li Y. et al., 2025; Li T. et al., 2025). Despite the global consensus on the elimination of cervical cancer and the promotion of prevention and control, there are still many challenges: there are significant inequalities in HPV vaccination, cervical cancer screening, and standardized treatment accessibility in different countries and regions, and the progress of low- and middle-income countries is lagging behind; limited access to medical services, differences in cultural concepts, and unequal distribution of resources also restrict the promotion of prevention and control. In addition, the existing research on the quantitative analysis of prevention and control effects in different regions and the optimization of prevention and control measures still need to be deepened.

In-depth analysis of the molecular regulation mechanism of the occurrence and development of cervical cancer is the key to the development of new precision treatment strategies. Tumor angiogenesis is the core link of tumor growth and metastasis. Vascular endothelial growth factor (VEGF) pathway and Notch pathway, as the core signal network regulating this process, play a key role in cancer progression (Vimalraj, 2022; Zhang et al., 2025). The VEGF pathway is the core regulator of angiogenesis. Its family members (such as VEGF-A, VEGF-C, etc.) initiate downstream PI3K, RAS/RAF/ERK signaling cascades by binding to receptors such as VEGFR-2, significantly promote the proliferation, migration and differentiation of endothelial cells, and provide the necessary oxygen and nutritional support for tumors. Drugs targeting this pathway (such as bevacizumab, ranibizumab, etc.) have been approved for use in a variety of tumors such as metastatic cervical cancer, confirming their therapeutic potential (Sun et al., 2025; Wang et al., 2024; Ma et al., 2019; Zhou et al., 2022). The Notch pathway, through the interaction of receptors (Notch1-4) and ligands (Dll1, Dll4, etc.), not only triggers the expression of cyclins, accelerates cell proliferation, and inhibits apoptosis, but also deeply participates in the fine-tuning of angiogenesis, affecting the formation of vascular patterns and the differentiation of arteries and veins. Meanwhile, Notch has a complex interplay with the inflammatory mediator NF-κB, which can further activate the expression of VEGF, creating a cancer-promoting signal amplification effect (Wang J. et al., 2025; Limones-Gonzalez et al., 2024). There is a close cross-regulation and synergy between VEGF pathway and Notch pathway in the angiogenesis and growth of various solid tumors. When VEGF binds to VEGFR-2, it can activate the expression of Dll4 in Notch pathway. Dll4 then binds to Notch1/4 to further activate the Notch signaling pathway. The activated Notch pathway regulates the VEGF pathway through negative feedback and reduces the expression of VEGFR-2, thereby inhibiting the proliferation, migration and vascular germination of endothelial cells, which together constitute a complex signal network to drive the malignant evolution of tumor (Saleh et al., 2025). In cervical cancer, the abnormal activation of these pathways may also be involved in tumor invasion and metastasis. The in-depth interpretation of their interaction mechanisms is expected to provide new molecular targets for targeted therapy of cervical cancer, and provide important scientific support for improving the treatment dilemma of patients in low-and middle-income areas and optimizing global prevention and control strategies.

As a medicinal resource with regional characteristics in Xinjiang, Ferula plants show unique scientific research value and application potential in the field of antitumor research (Wariss et al., 2026). There are about 150 species of this genus in the world, and 31 species are distributed in China. Among them, 25 species are exclusively distributed in Xinjiang, and the resource richness is extremely high. Among many Ferula plants, *Ferula songorica* Pall. ex Spreng has become a research hotspot due to its considerable reserves. Our previous work showed that the ethanol extract of *F. songorica* (FSEE) produced strong antitumor effects in mice bearing cervical cancer xenografts at doses of 0.75 g/kg and 1.5 g/kg, yielding tumor inhibition rates of 72.76% and 88.67%, respectively (Liu et al., 2023). In zebrafish embryos, FSEE at 10 μg/mL inhibited intersegmental angiogenesis by 50.12%, confirming its anti-angiogenic activity (Yilimire et al., 2023). Through further separation and purification, we isolated the plant metabolite akiferidin from *F. songorica*. Preliminary data indicate that akiferidin markedly inhibits proliferation and migration of U14 cervical cancer cells, with an IC_50_ of 8.06 μg/mL for proliferation, suggesting potential to impede tumor progression and pathological angiogenesis.

Akiferidin has shown promising antitumor and anti-angiogenic activity in preliminary studies, and the VEGF/DLL4-Notch pathway critically regulates tumor growth and pathological angiogenesis. This study therefore systematically evaluates akiferidin’s *in vitro* antitumor and anti-angiogenic effects and further assesses its *in vivo* antitumor efficacy and mechanisms in a cervical cancer xenograft mouse model, focusing on whether akiferidin acts by modulating the VEGF/DLL4-Notch signaling pathway. The results will provide key experimental evidence for developing novel natural anti-cervical cancer agents and are expected to support translational research on antitumor drugs derived from Xinjiang-specific medicinal materials.

## 2 Materials and methods

### 2.1 Plant materials and extraction procedure

*F. songorica* was collected from the northern mountainous area of Tacheng, Xinjiang. It was identified by Chief Traditional Chinese Medicine Pharmacist Li Yonghe from the Xinjiang Autonomous Region Traditional Chinese Medicine Hospital as consistent with the description of the species in the Flora of China. The roots of *F. songorica* were cleaned of mud and impurities, dried in a cool and ventilated place, and then ground into coarse powder. 10.0 g of the coarse powder of *F. songorica* was weighed, and 30 mL of 95% ethanol was added for ultrasonic extraction five times. The filtrate solution was filtered, combined, concentrated under reduced pressure, dried, and 3.36 g of *F. songorica* ethanol extract (FSEE) was obtained.

### 2.2 Determination of the content of the active plant metabolite akiferidin in the ethanol extract of F. songorica

Weigh precisely 10 mg of the ethanol extract of *F. songorica* and dissolve it in methanol to a volume of 10 mL to prepare a 1 mg/mL solution. Filter the solution through a 0.45 μm microporous membrane to obtain the test sample solution. Weigh precisely 1 mg of the standard akiferidin and dissolve it in methanol to a volume of 10 mL to prepare a 0.1 mg/mL solution. Filter the solution through a 0.45 μm microporous membrane to obtain the akiferidin reference solution. The chromatographic conditions are as follows: XTERRA MS® C18 analytical column (4.6 × 250 mm, 5 μm), mobile phase: acetonitrile and 0.1% phosphoric acid water, gradient elution: 0–35 min: 52% A- 60% A, 35–43 min: 60% A- 88% A, 43–45 min: 88% A- 52% A, 45–55 min: 52% A- 52% A, flow rate: 1 mL/min, column temperature: 30 °C, detection wavelength: 266 nm, injection volume: 10 μL. The content of akiferidin is calculated based on the calibration curve (akiferidin, Y = 2057x - 96.38, *R* ^2^ = 0.9999, linear range: 30 ng-3 μg). The precision, repeatability, solution stability, and recovery rate of the test sample solution are examined methodologically.

### 2.3 MTT assay

U14 cervical cancer cells (Institute of Basic Medical Sciences, Chinese Academy of Medical Sciences, Beijing, China) were seeded into 96-well plates at 2 × 10^5^ cells/mL. After 24 h of culture, 200 μL akiferidin was added to each well at concentrations of 200 μg/mL, 100 μg/mL, 50 μg/mL, 25 μg/mL, 12.5 μg/mL, and 6.25 μg/mL. The cells were incubated for an additional 24 h, after which 100 μL MTT was added to each well. Following 3–4 h of incubation, 150 μL DMSO was added and the plates were shaken for 15 min. Optical density (OD) at 490 nm was measured with a microplate reader (Multiskan Spectrum, Thermo Scientific, United States).

### 2.4 Wound healing assay

U14 cells were plated in 6-well plates at 1 × 10^5^ cells/mL. Using an autoclaved 10 μL sterile pipette tip, each well was scratched lightly three times. The scratch distance (width between scratch lines) at 0 h was recorded with an inverted microscope (IX-71, Olympus, Tokyo, Japan). Akiferidin (1, 2, and 4 μg/mL) and the positive control paclitaxel (10 nM; Sichuan Huiyu Pharmaceutical Co., Ltd., Ziyang, China; Lot No. 120225006) (Jiangsu Hansoh Pharmaceutical Group Co., Ltd., China) were added to the wells. After 48 h of incubation, the scratch distance was measured again.

### 2.5 Transwell assay

U14 cells were seeded in 6-well plates at 1 × 10^5^ cells/mL. Akiferidin (1, 2, and 4 μg/mL) or paclitaxel (10 nM) was added and cells were incubated for 48 h. Cells were then collected, resuspended in serum-free medium, and seeded into the upper chamber of Transwell inserts (200 μL per well). For invasion assays, the upper chambers were precoated with Matrigel. The lower chambers received 200 μL medium containing 10% fetal bovine serum. After an additional 48 h incubation, cells on the lower surface were fixed with 4% paraformaldehyde and stained with crystal violet for 30 min invaded cells were then counted.

### 2.6 Tube formation assay

Matrigel was thawed on ice at 4 °C overnight and dispensed into a prechilled 96-well plate at 50 μL per well. The plate was incubated at 37 °C with 5% CO_2_ for 30 min to allow complete polymerization of the matrix. Human umbilical vein endothelial cells (HUVECs, Institute of Basic Medical Sciences, Chinese Academy of Medical Sciences, Beijing, China) were harvested and resuspended in endothelial cell medium (ECM) to prepare a single-cell suspension adjusted to a final density of 2 × 10^5^ cells/mL. Cell suspensions were allocated and treated as follows: the blank control group received blank ECM; the positive control group received bevacizumab at a final concentration of 100 μg/mL; experimental groups received akiferidin at final concentrations of 1, 2, and 4 μg/mL. These mixtures were seeded onto the polymerized Matrigel at 110 μL per well, with three technical replicates per group. After 6 h incubation at 37 °C with 5% CO_2_, capillary-like tube formation was examined by inverted phase-contrast microscopy. Six nonoverlapping random fields were captured per well, and tube formation parameters were quantified using ImageJ.

### 2.7 Experimental animals

Specific-pathogen-free (SPF) female C57BL/6 mice (6–8 weeks old, 20.0 ± 2.0 g) were acclimated for 7 days at 23 °C ± 2 °C, 55% ± 5% relative humidity, and a 12 h light/12 h dark cycle with *ad libitum* access to food and water. U14 cervical cancer cells in log phase were detached with 0.25% trypsin-EDTA, washed twice in sterile 0.9% saline, and adjusted to 2 × 10^6^ cells/mL. After anesthesia by isoflurane inhalation (RWD Life Science Co., Ltd., Shenzhen, China; Lot No. 217180501), each mouse received a sterile subcutaneous abdominal inoculation of 0.2 mL of the cell suspension (4 × 10^5^ cells). Mice were monitored daily and randomized into groups when tumor volumes reached 100–150 mm^3^ (approximately 7–10 days postinoculation). All *in vivo* experiments were performed in two independent cohorts, and treatments were administered once daily by intraperitoneal (i.p.) injection for the study duration. In the first cohort, animals were randomized into six groups (10 mice per group): blank control, tumor-bearing model, akiferidin treatment groups (10, 15, and 20 mg/kg; the dose range was determined based on the *in vitro* IC_50_ value (8.06 μg/mL) of akiferidin against U14 cells, combined with pre-experimental *in vivo* safety evaluation and dose-dependent anti-tumor efficacy screening. The 20 mg/kg dose was selected as the optimal dose for subsequent combination experiments, as it exhibited the most significant anti-tumor efficacy without observable systemic toxicity in tumor-bearing mice), and a paclitaxel positive control group (15 mg/kg; this dose was selected as it is a classic and widely validated positive control dose in U14 cervical cancer xenograft mouse models, which can achieve significant anti-tumor efficacy without excessive lethal toxicity in immunocompetent C57BL/6 mice). The blank control and model groups received the same volume of the corresponding vehicle on the identical dosing schedule. For mechanistic validation, animals were randomized into five groups (10 mice per group): model group, akiferidin monotherapy group (20 mg/kg), paclitaxel group, bevacizumab (VEGF inhibitor, 1 mg/kg; Innovent Biologics, Inc., Suzhou, China; Lot No. A202510003) group, and akiferidin plus bevacizumab combination group.

The animal study was reviewed and approved by the Animal Ethics Committee of Xinjiang Medical University (Protocol No. IACUC-2026012305).

### 2.8 *In vivo* imaging system (IVIS)

Mice were injected intraperitoneally with D-luciferin (150 mg/kg; Caliper Life Sciences, Hopkinton, MA, United States) and, 10 min later, maintained under anesthesia with 2% isoflurane. Each mouse was placed supine and secured on the imaging platform with the tumor-bearing abdomen facing the lens of the Xenogen IVIS 200 bioluminescence imaging system (Caliper Life Sciences, Hopkinton, MA, United States), and images were acquired with a 10-s exposure. Using Living Image 2.5 software, a region of interest (ROI) was drawn over the tumor to automatically calculate and record the total radiant efficiency (Total Radiant Efficiency, photons/second/cm^2^/Sr), which was used to assess tumor burden and drug efficacy.

### 2.9 Tissue sampling from animals and H&E staining

Mice in each group received continuous treatment for 12 days. Twenty-four hours after the final dose, the mice were sacrificed under anesthesia. Tumors, spleens, kidneys, and thymus were rapidly dissected. After blotting surface moisture from the tissues with filter paper, their weights were measured and recorded. Tumor specimens were handled in two ways: one portion was fixed in 10% neutral formalin, and the other was stored at −70 °C in an ultra-low-temperature freezer for later analysis. Formalin-fixed tumors were dehydrated through graded ethanol, embedded in paraffin, sectioned, and stained with hematoxylin and eosin (H&E). Pathological features were examined and analyzed by light microscopy.

### 2.10 Monitoring of liver and kidney function

After blood collection for each mouse group, whole blood was placed in a 1.5 mL centrifuge tube and allowed to clot at room temperature for 30–60 min. The sample was then centrifuged at 4 °C and 14,000 × g for 10–15 min. The supernatant serum was aspirated with a pipette and transferred to a new tube. To remove residual erythrocytes, the serum may be centrifuged again at 4 °C and 14,000 × g for 2–3 min, and the resulting supernatant transferred to a clean tube. Serum liver and kidney function indicators were measured using an automatic biochemical analyzer.

### 2.11 Immunofluorescence

Tumor tissues were fixed in 10% neutral formalin, embedded in paraffin, and sectioned at 5 μm. Sections were dewaxed in xylene (2 × 10 min), rehydrated through graded ethanol (100%, 95%, 85%, 75%) for 5 min each, and rinsed twice with distilled water. Antigen retrieval was performed in EDTA repair solution (pH 9.0) in a microwave at medium power for 20 min, and sections were allowed to cool at room temperature. After three washes with PBS (5 min each), the tissue borders were circled, and sections were blocked with 3% BSA for 30 min; the blocking solution was then removed. Sections were incubated overnight at 4 °C in a humidified chamber with CD31 primary antibody (Abcam, Cambridge, United Kingdom; Cat. No. ab28364; diluted 1:50 in PBS). Following three PBS washes (5 min each), Alexa Fluor® 488 secondary antibody (diluted 1:500) was applied and incubated in the dark for 1 h. Nuclei were counterstained with DAPI for 10 min, followed by three PBS washes. Sections were mounted with an anti-quenching medium and imaged on a fluorescence microscope (excitation 488 nm; emission 515–555 nm). For each sample, five random high-power fields (×200) were acquired, and the proportion of CD31-positive area was quantified with ImageJ to assess vascular density.

### 2.12 Immunohistochemistry (IHC)

Tumor tissues were fixed in 10% neutral formalin, embedded in paraffin, and sectioned at 5 μm. Sections were dewaxed twice in xylene (10 min each), rehydrated through graded ethanol (100%, 95%, 85%, 75%; 5 min each), and rinsed twice in distilled water. Antigen retrieval was performed in EDTA buffer (pH 9.0) in a microwave at medium power for 20 min, followed by natural cooling. Sections were washed three times in PBS (5 min each) and treated with 3% hydrogen peroxide at room temperature for 25 min to quench endogenous peroxidase, then washed three times in PBS. After blocking with 3% BSA for 30 min, sections were incubated overnight at 4 °C with primary antibodies against VEGF-A (Abcam, Cambridge, United Kingdom; Cat. No. ab46154; 1:100), VEGFR-2 (Abcam, Cambridge, United Kingdom; Cat. No. ab315238; 1:50), DLL4 (Abcam, Cambridge, United Kingdom; Cat. No. ab183532; 1:100), and Notch4 (Abcam, Cambridge, United Kingdom; Cat. No. ab184742; 1:200). Following PBS washes, HRP-conjugated secondary antibodies (1:500) were applied at room temperature for 50 min and then washed with PBS. Sections were developed in DAB chromogenic solution until a brown-yellow color appeared, then the reaction was stopped with tap water. Nuclei were counterstained with hematoxylin for 3 min, differentiated in 1% hydrochloric acid ethanol for 5 s, and treated with a bluing solution for 10 min, followed by rinsing in running water. Slides were dehydrated through graded ethanol (75%→100%), cleared in xylene, and coverslipped with neutral mounting medium. Images were acquired with an optical microscope (×400). The mean optical density (MOD) of positive areas was measured using Image-Pro Plus software to quantify protein expression intensity.

### 2.13 Western blotting analyses

Tumor tissues were pulverized in liquid nitrogen using a low-temperature tissue grinder, and total protein was extracted with RIPA lysis buffer supplemented with 1 mM PMSF and phosphatase inhibitors. Lysates were mixed and incubated on ice at 4 °C for 30 min, then centrifuged at 12,000 *g* for 10 min at 4 °C to collect the protein-containing supernatant. Total protein concentration for each sample was determined with a BCA protein assay kit. Equal amounts (30 μg) of protein from each sample were resolved by 10% sodium dodecyl sulfate–polyacrylamide gel electrophoresis (SDS-PAGE) and transferred to polyvinylidene fluoride (PVDF) membranes at a constant current of 200 mA. After transfer, membranes were blocked with 5% (w/v) nonfat dry milk in TBST at room temperature for 1 h to prevent nonspecific binding. Membranes were incubated overnight at 4 °C with the following primary antibodies: VEGF-A (Abcam, Cambridge, United Kingdom; Cat. No. ab46154; 1:1000), VEGFR-1 (Abcam, Cambridge, United Kingdom; Cat. No. ab32152; 1:1000), VEGFR-2 (Abcam, Cambridge, United Kingdom; Cat. No. ab315238; 1:1000), DLL4 (Abcam, Cambridge, United Kingdom; Cat. No. ab183532; 1:2000), Notch1 (Abcam, Cambridge, United Kingdom; Cat. No. ab52627; 1:2000), Notch4 (Abcam, Cambridge, United Kingdom; Cat. No. ab184742; 1:1000), and CD2AP (Invitrogen, Carlsbad, CA, United States of America; Cat. No. PA5-51894; 1:600). After primary antibody incubation, membranes were washed three times with TBST (10 min each) and then incubated with HRP-conjugated secondary antibodies (1:5000) at room temperature for 1 h. Membranes were washed three times with TBST (10 min each), then incubated for 1 min before development with an enhanced chemiluminescence (ECL) kit. Chemiluminescent signals were captured using a digital chemiluminescence imaging system. For quantification, grayscale intensity of each target band was measured with ImageJ software (National Institutes of Health, Bethesda, MD, United States of America), and the background signal was subtracted from each measurement. Relative expression of each target protein was calculated by normalizing its grayscale intensity to the internal reference GAPDH from the same sample to account for uneven loading. All Western blot experiments were performed independently in triplicate, yielding consistent results.

### 2.14 Molecular docking and molecular dynamics simulation

Docking of VEGF-A, VEGFR-1, and VEGFR-2 with akiferidin was carried out following a standard preparation and docking workflow. First, macromolecular structures were prepared. The three-dimensional structure of VEGF-A was retrieved from the Protein Data Bank (PDB), and a human-derived crystal structure with resolution <3 was selected and saved in PDB format (Burley et al., 2023). The structure was loaded into PyMOL to remove water and ligand molecules from the crystal. The cleaned structure was then imported into AutoDock to add all hydrogens and assign charges, and the resulting file was saved in pdbqt format. Next, the small-molecule structure of akiferidin was obtained from PubChem, standardized in OpenBabel, and imported into AutoDock to generate its rigid conformation; the ligand was saved in pdbqt format. Grid-box dimensions, defined by X, Y, and Z coordinates, were set to encompass the protein active sites. Finally, molecular docking was performed with AutoDock Vina to determine the binding affinities of akiferidin for VEGF-A, VEGFR-1, and VEGFR-2.

Molecular dynamics simulations were conducted for the akiferidin–VEGF-A complex. The simulation system was built with GROMACS and parameterized with the CHARMM force field to describe intermolecular interactions (Valdés-Tresanco et al., 2021). Energy minimization removed unfavorable atomic contacts and steric clashes. The system was then equilibrated in NVT and NPT ensembles to stabilize temperature and pressure. Production simulations were run for 100 ns, during which molecular trajectories and relevant system parameters were recorded (Kim et al., 2017). Analysis of these trajectories yielded details on structural changes, binding stability, and intermolecular interaction energies of the akiferidin–VEGF-A complex. These results provide a molecular-level understanding of the interaction mechanism between akiferidin and VEGF-A.

### 2.15 Statistical analysis

Data were expressed as the means ± standard error of the means (SEM). The statistical significance of the differences was assessed by ANOVA followed by *post hoc* test with LSD method. *P* values less than 0.05 were considered statistically significant.

## 3 Results and discussion

### 3.1 Determination of akiferidin content and methodological investigation

**FIGURE 1:**
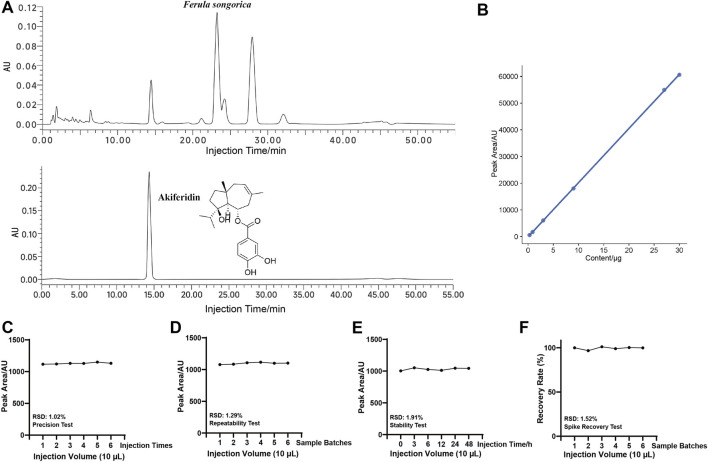
The HPLC analysis and methodological validation of akiferidin in FSEE. **(A)** HPLC chromatograms of *F*. *songorica* solution and akiferidin; **(B)** Linearity graph of akiferidin reference substance; **(C)** Precision test; **(D)** Repeatability test; **(E)** Stability test; **(F)** Recovery test.

Akiferidin was identified as the characteristic constituent of FSEE. We developed and validated a high-performance liquid chromatography (HPLC) method to quantify akiferidin in FSEE (Figure 1). Instrument precision was evaluated by six consecutive injections of the same sample solution, yielding a relative standard deviation (RSD) of 1.02%, which met the predefined acceptance criteria. Method repeatability was assessed by parallel analysis of six independent sample batches and produced an RSD of 1.29%, within the acceptable range for repeatability. Akiferidin stability in the test solution was monitored at ambient temperature over 48 h, with measurements at 0, 3, 6, 12, 24, and 48 h; the RSD of peak areas was 1.91%, indicating chemical stability in the test matrix throughout the observation period. Method accuracy was confirmed by a spike-and-recovery assay in which six samples were prepared by adding 1 mL of akiferidin reference standard solution (0.4 mg/mL) to 1 mL of FSEE test solution (0.01 g/mL); mean recovery was 99.48% with an RSD of 1.52%. All validation parameters complied with the acceptable limits specified in international guidelines for quantitative analytical methods, demonstrating that the HPLC method is robust, accurate, and reliable for quantifying akiferidin in FSEE. Using the validated method, akiferidin content in FSEE was determined to be 57.45 ± 1.42 mg/g.

### 3.2 *In vitro* antitumor and anti-angiogenic activities of akiferidin

**FIGURE 2:**
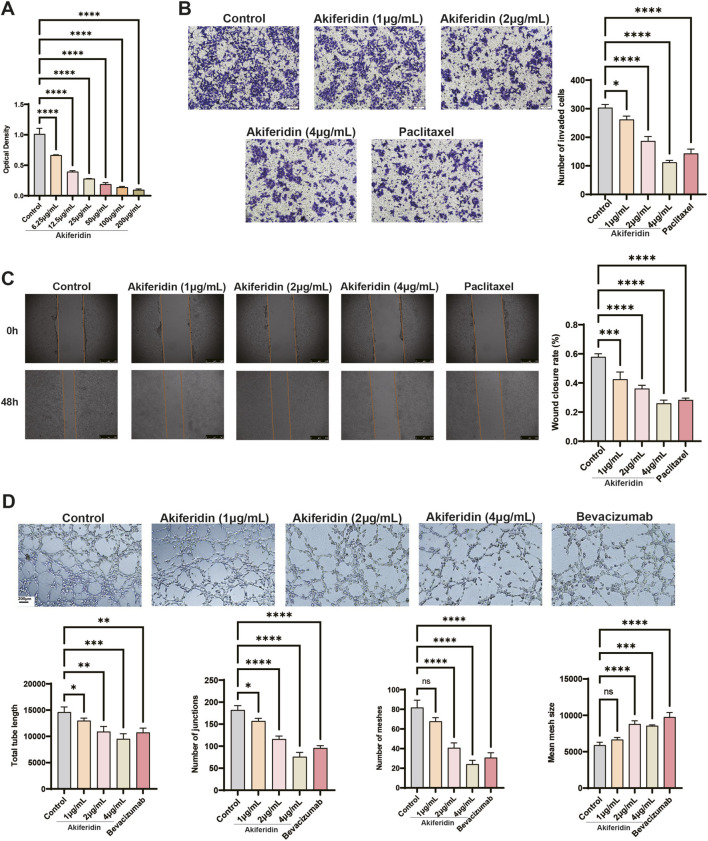
Inhibitory effects of akiferidin on tumor cell malignant phenotypes and angiogenesis *in vitro*. **(A)** Viability of U14 cells treated with gradient concentrations of akiferidin detected by MTT assay. **(B)** Invasion ability of U14 cells detected by Transwell assay and its quantitative analysis. **(C)** Migration ability of U14 cells detected by wound healing assay and its quantitative analysis of 48 h wound closure rate. **(D)** Tube formation of HUVECs and quantitative analysis of core angiogenesis indicators. *P < 0.05, **P < 0.01, ***P < 0.001, ****P < 0.0001; ns, no significant difference.

To elucidate the *in vitro* antitumor and anti-angiogenic activities of akiferidin, we systematically assessed its effects on U14 cervical cancer cell proliferation, invasion, and migration, and on tube formation by HUVECs, using MTT colorimetric, Transwell invasion, wound-healing, and Matrigel-based tube formation assays. The MTT assay showed that akiferidin inhibited U14 cell proliferation in a concentration-dependent manner over the range 6.25–200 μg/mL. Relative to the control group, all gradient concentrations of akiferidin significantly reduced U14 cell viability (all *P* < 0.0001, Figure 2A), with a calculated proliferation inhibition IC_50_ of 8.06 μg/mL. The Transwell invasion assay indicated that akiferidin suppressed U14 cell invasiveness in a concentration-dependent fashion. At 1 μg/mL, akiferidin significantly reduced the number of transmembrane cells (*P* < 0.05), and the inhibitory effects were further strengthened at 2 μg/mL and 4 μg/mL, with all differences reaching extreme significance (all *P* < 0.0001, Figure 2B). The wound healing assay showed that akiferidin inhibited U14 cell lateral migration in a concentration-dependent manner. After 48 h of treatment, akiferidin at 1 μg/mL produced an extremely significant reduction in wound closure (*P* < 0.001), and the effects at 2 μg/mL and 4 μg/mL were even more pronounced (all *P* < 0.0001, Figure 2C). In the HUVEC tube formation assay, control cultures formed intact, dense capillary-like networks, whereas akiferidin disrupted tubular integrity and inhibited *in vitro* angiogenesis in a concentration-dependent fashion (Figure 2D). Quantitatively, akiferidin at 1 μg/mL significantly decreased total tube length and reduced the number of junctions (all *P* < 0.05), but did not affect the number of closed meshes or average mesh area (all *P* > 0.05, ns). Akiferidin at 2 μg/mL and 4 μg/mL produced significant to highly significant changes across all four core tube-formation metrics (*P* < 0.01 to *P* < 0.0001). Together, these findings indicate that akiferidin has notable *in vitro* antitumor and anti-angiogenic activity.

### 3.3 *In vivo* anti-tumor efficacy of akiferidin in cervical cancer xenograft-bearing mice

**FIGURE 3:**
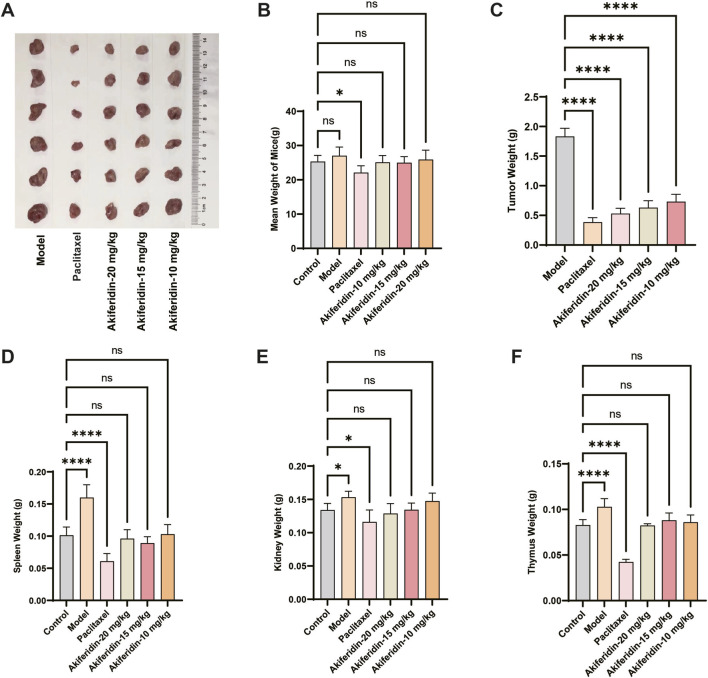
Antitumor activity of akiferidin on cervical cancer xenograft-bearing mice. **(A)** Photographs of tumors from cervical cancer xenograft-bearing mice in each group. **(B)** Body weights of mice in each group. **(C)** Tumor weights of mice in each group. **(D)** Spleen weights of mice in each group. **(E)** Kidney weights of mice in each group. **(F)** Thymus weights of mice in each group. *P < 0.05, **P < 0.01, ***P < 0.001, and ****P < 0.0001.

To evaluate the *in vivo* antitumor efficacy and safety of akiferidin, we established a U14 cervical cancer–bearing mouse model and used the clinical first-line chemotherapeutic paclitaxel as a positive control. We then assessed the effects of varying akiferidin doses on tumor growth, body weight, and major organs. Gross tumor images (Figure 3A) showed large, well-established tumors in the model group. Tumor volumes were markedly reduced in the paclitaxel group and in all akiferidin dose groups, confirming akiferidin’s *in vivo* antitumor activity. Body weight monitoring (Figure 3B) revealed a modest but statistically significant reduction only in the paclitaxel group compared with the blank control (*P* < 0.05), whereas no significant differences were observed among the akiferidin dose groups (*P* > 0.05), indicating no obvious systemic toxicity. Quantitative analysis of tumor weight (Figure 3C) showed that tumor weight in the model group was significantly higher than in all treatment groups (*P* < 0.0001). Akiferidin at 10, 15, and 20 mg/kg each produced an extremely significant reduction in tumor weight (all *P* < 0.0001), with the antitumor effect exhibiting dose dependence.

Organ weight analysis showed that the spleen (Figure 3D), kidney (Figure 3E), and thymus (Figure 3F) of tumor-bearing model mice were significantly heavier than those of the blank control group (*P* < 0.05 or *P* < 0.0001), indicating tumor-induced pathological changes. Treatment with the positive control paclitaxel markedly reduced spleen and thymus weights relative to the model group, and these organ weights were significantly lower than those of the blank control group (*P* < 0.0001), reflecting clear toxic effects on immune organs. By contrast, all akiferidin dose groups normalized the tumor-associated increases in organ weight, returning spleen, kidney, and thymus weights to values close to those of the blank control group, with no statistical differences versus control (*P* > 0.05). These findings indicate that akiferidin caused no obvious toxicity to major or immune organs in mice and demonstrated favorable *in vivo* safety. In conclusion, akiferidin exhibits significant inhibitory activity against U14 cervical cancer *in vivo* and shows substantially better safety than the conventional chemotherapeutic paclitaxel, with no evident systemic toxicity or immune organ damage, thereby providing a solid pharmacodynamic foundation for further mechanistic studies and therapeutic development.

### 3.4 Enhanced anti-tumor efficacy of akiferidin in combination with bevacizumab

**FIGURE 4:**
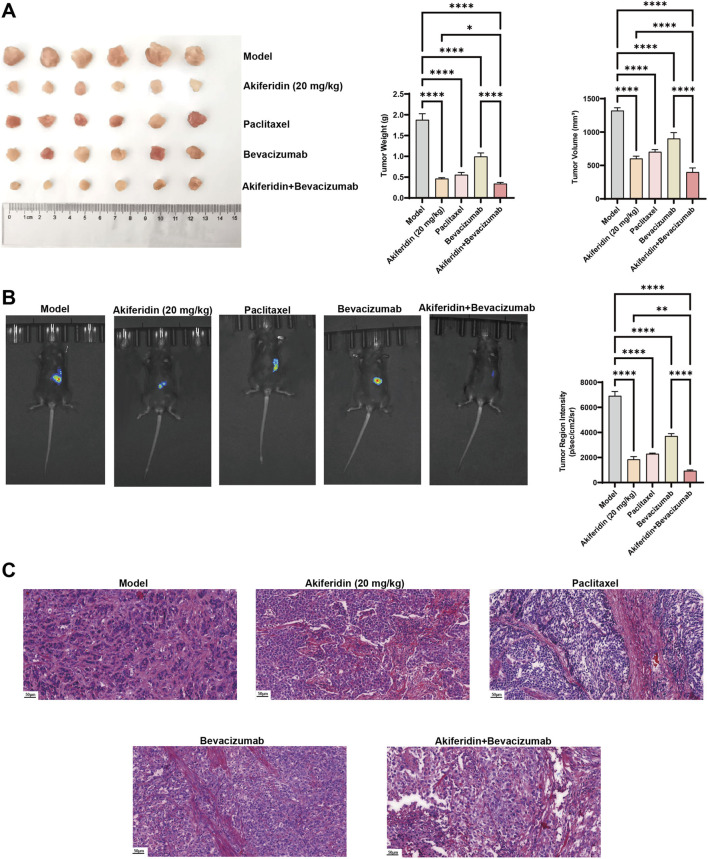
Antitumor effects of akiferidin as a monotherapy and in combination on cervical cancer xenograft-bearing mice. **(A)** Photographs of tumors and bar charts of tumor weights and volumes of cervical cancer xenograft-bearing mice in each group. **(B)** *In vivo* imaging of tumors and bar charts of tumor fluorescence intensity of mice in each group. **(C)** H&E stained pathological sections of tumor tissues in each group. *P < 0.05, **P < 0.01, ***P < 0.001, and ****P < 0.0001.

Given akiferidin’s confirmed antitumor activity as a monotherapy in cervical cancer xenograft models, this study compared the antitumor effects of akiferidin (20 mg/kg) alone, akiferidin combined with bevacizumab, and paclitaxel or bevacizumab alone. All treatment groups—akiferidin monotherapy, paclitaxel, bevacizumab, and the akiferidin plus bevacizumab combination—showed markedly reduced tumor volumes relative to the model group (Figure 4A). Quantitative analysis confirmed that tumor weights and volumes in these treatment groups were significantly lower than those in the model group (*P* < 0.0001). Moreover, compared with the bevacizumab monotherapy group, the co-administration of akiferidin and bevacizumab exhibited a significantly more potent anti-tumor effect (*P* < 0.0001), indicating a favorable combination effect of the two agents.

The *in vivo* imaging of tumor-bearing mice (Figure 4B) showed that tumor fluorescence signals, which reflect tumor burden, were markedly weaker in all treatment groups than in the model group. Quantitative analysis confirmed a significant reduction in tumor fluorescence intensity in each treatment group (*P* < 0.0001), indicating that these treatment regimens effectively inhibited tumor growth. H&E-stained sections of tumor tissue (Figure 4C) revealed that tumor cells in the model group were densely packed and actively proliferating. However, the tumor tissues in the treatment groups all showed varying degrees of cell necrosis and reduced cell density, with the most significant pathological changes observed in the combination group of akiferidin and bevacizumab.

### 3.5 Monitoring of liver and kidney function

**FIGURE 5:**
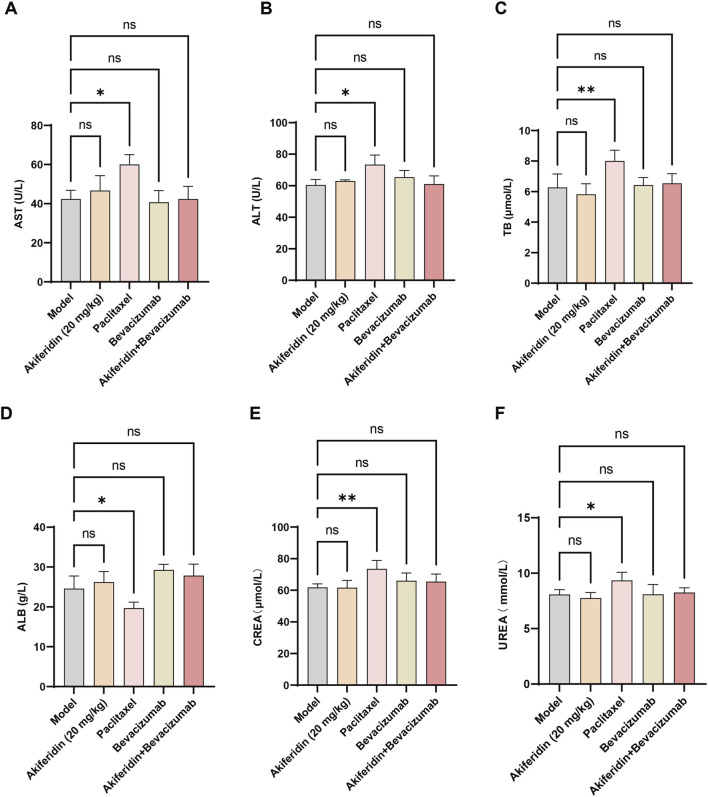
Effects of akiferidin as a monotherapy and in combination on serum biochemical indicators in cervical cancer xenograft-bearing mice. **(A)** Serum aspartate aminotransferase (AST) levels in each group of tumor-bearing mice. **(B)** Serum alanine aminotransferase (ALT) levels in each group of tumor-bearing mice. **(C)** Serum total bilirubin (TB) levels in each group of tumor-bearing mice. **(D)** Serum albumin (ALB) levels in each group of tumor-bearing mice. **(E)** Serum creatinine (CREA) levels in each group of tumor-bearing mice. **(F)** Serum urea (UREA) levels in each group of tumor-bearing mice.

To assess the *in vivo* safety of akiferidin alone and combined with bevacizumab, we measured serum biochemical markers of liver function, protein metabolism, and renal function in tumor-bearing mice to determine the regimens’ effects on major organ function. First, we examined liver-related markers. As shown in Figures 5A–C, serum aspartate aminotransferase (AST, Figure 5A), alanine aminotransferase (ALT, Figure 5B), and total bilirubin (TB, Figure 5C) in the akiferidin monotherapy, bevacizumab monotherapy, and akiferidin plus bevacizumab groups did not differ significantly from the model group (all *P* > 0.05, ns). By contrast, the positive-control paclitaxel group showed significant increases in AST (*P* < 0.05), ALT (*P* < 0.05), and TB (*P* < 0.01) versus the model group. These data indicate that akiferidin, alone or combined with bevacizumab, does not cause abnormal liver dysfunction in tumor-bearing mice.

We next assessed indicators of protein metabolism. As shown in Figure 5D, serum albumin (ALB) was significantly lower in the paclitaxel group than in the model group (*P* < 0.05). ALB levels in the akiferidin monotherapy, bevacizumab monotherapy, and combination groups did not differ significantly from the model group (all *P* > 0.05, ns). These results indicate that akiferidin-containing regimens did not disturb systemic protein metabolism in tumor-bearing mice. Finally, we evaluated renal function. As shown in Figures 5E,F, serum creatinine (CREA, Figure 5E) and urea (UREA, Figure 5F) in the akiferidin monotherapy, bevacizumab monotherapy, and combination groups showed no significant differences versus the model group (all *P* > 0.05, ns). By contrast, the paclitaxel group exhibited significant increases in both CREA (*P* < 0.01) and UREA (*P* < 0.05) relative to the model group. These data confirm that akiferidin and its combination regimen did not induce notable renal injury in tumor-bearing mice. In summary, akiferidin monotherapy and the akiferidin–bevacizumab combination demonstrated favorable *in vivo* safety, with no adverse effects on liver function, protein metabolism, or renal function in tumor-bearing mice.

### 3.6 Detection of tumor vascular density

**FIGURE 6:**
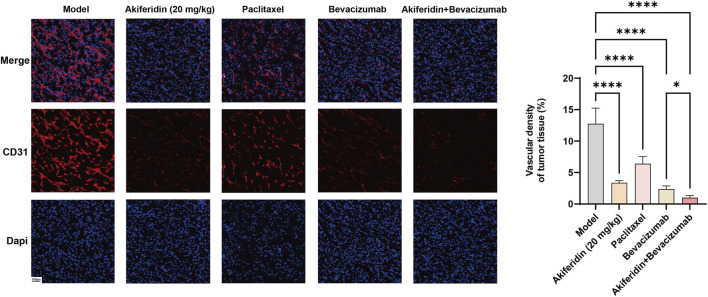
Effects of akiferidin as a monotherapy and in combination on tumor tissue vascular density (red for endothelial cells, blue for nuclei),*P < 0.05,****P < 0.0001.

Angiogenesis is a central pathological driver of tumor growth and invasion. To assess the treatments’ anti-angiogenic effects, we measured microvascular density in tumor tissues. As shown in Figure 6, the model group exhibited the highest microvascular density (15.06% ± 0.96%). Compared with the model group, microvascular density decreased markedly in the akiferidin monotherapy group (1.83% ± 1.26%, *P* < 0.0001), the paclitaxel group (4.40% ± 1.09%, *P* < 0.0001), and the bevacizumab group (1.97% ± 0.97%, *P* < 0.0001). Notably, the bevacizumab plus akiferidin combination further reduced microvascular density to 0.89% ± 0.42% relative to bevacizumab monotherapy (*P* < 0.05). These results indicate that both bevacizumab and akiferidin can effectively inhibit tumor angiogenesis, and their combination can further enhance this effect.

### 3.7 Inhibitory effect of akiferidin alone and in combination with bevacizumab on the VEGF/DLL4-Notch signaling pathway

**FIGURE 7:**
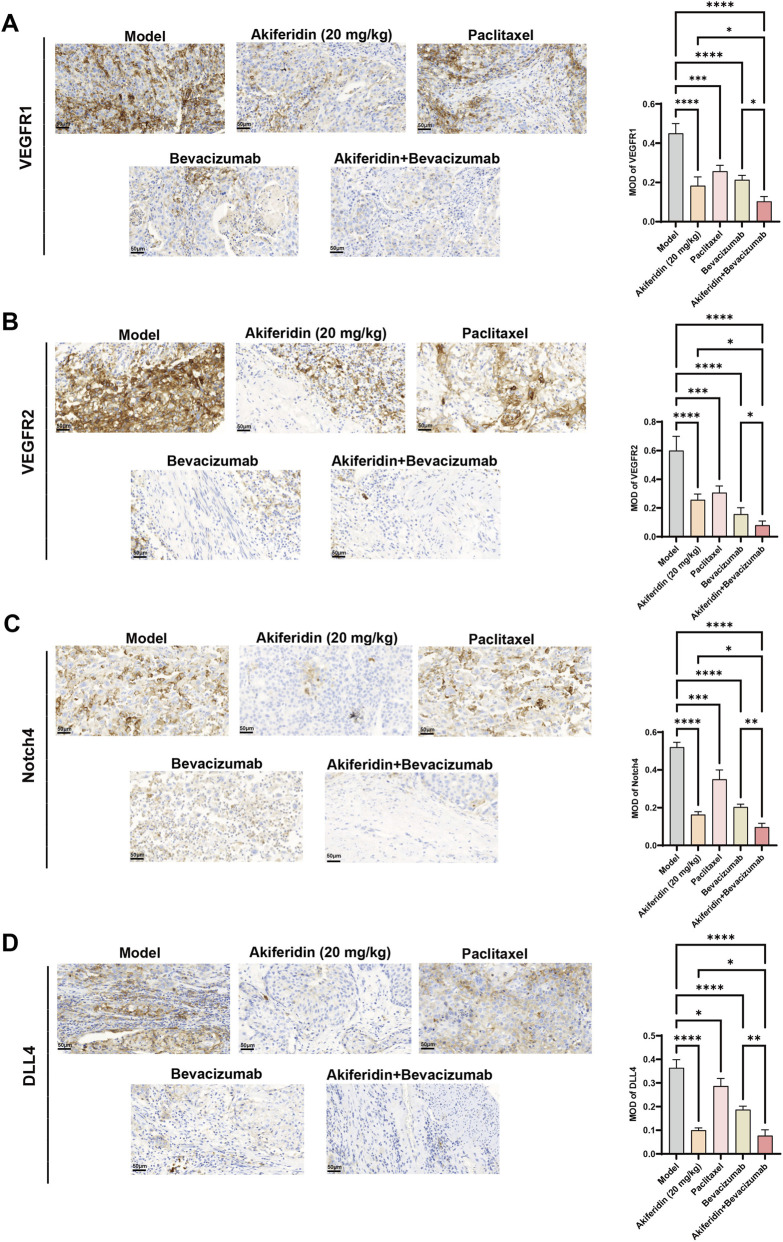
IHC detection of the effects of akiferidin on the expression of the VEGF/DLL4-Notch pathway in cervical cancer xenograft tissues. **(A)** IHC staining of VEGFR-1 in tumor tissues of mice in each group. **(B)** IHC staining of VEGFR-2 in tumor tissues of mice in each group. **(C)** IHC staining of Notch4 in tumor tissues of mice in each group. **(D)** IHC staining of DLL4 in tumor tissues of mice in each group. *P < 0.05, **P < 0.01, ***P < 0.001, ****P < 0.0001.

To elucidate the molecular mechanisms by which akiferidin regulates the VEGF/DLL4-Notch pathway to inhibit cervical cancer growth, we measured angiogenesis-related factors and key signaling proteins in tumor tissues using IHC and Western blot. IHC revealed strong positive staining and the highest mean optical density (MOD) values for VEGFR-1, VEGFR-2, Notch4, and DLL4 in the model group (Figures 7A–D), indicating marked activation of angiogenic signaling in the tumor-bearing state. Relative to the model group, akiferidin monotherapy, bevacizumab monotherapy, and the akiferidin + bevacizumab combination each produced highly significant reductions in VEGFR-1, VEGFR-2, Notch4, and DLL4 protein levels (all *P* < 0.0001). The paclitaxel group also significantly reduced VEGFR-1, VEGFR-2, and Notch4 (both *P* < 0.001) and decreased DLL4 levels (*P* < 0.05). Importantly, compared with bevacizumab alone, the akiferidin + bevacizumab combination more strongly inhibited VEGFR-1 and VEGFR-2 (both *P* < 0.05) and produced greater downregulation of DLL4 and Notch4 (both *P* < 0.01), indicating that the combination more effectively suppresses core VEGFR and Notch pathway proteins than bevacizumab monotherapy.

**FIGURE 8:**
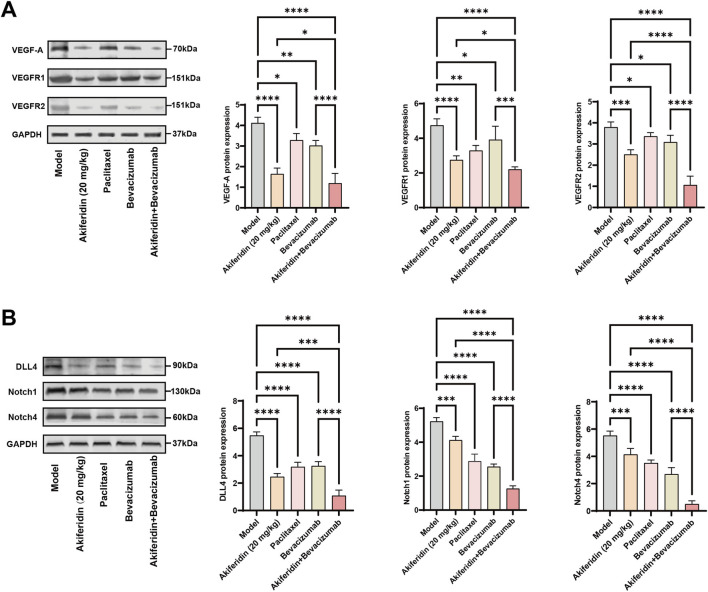
Effects of akiferidin on the expression of VEGF/DLL4-Notch signaling-related proteins in tumor tissues. **(A)** Representative Western blot bands and quantitative analysis of VEGF-A, VEGFR-1 and VEGFR-2. **(B)** Representative Western blot bands and quantitative analysis of DLL4, Notch1 and Notch4. GAPDH was used as the loading control. *P < 0.05, **P < 0.01, ***P < 0.001, ****P < 0.0001.

Western blot analysis confirmed and extended the above findings. As shown in Figure 8A, VEGF-A, VEGFR-1, and VEGFR-2 were highly expressed in tumor tissues from the model group, and all treatment groups significantly reduced the protein levels of these molecules (*P* < 0.05). Notably, the combination of akiferidin and bevacizumab produced a significantly greater suppression of VEGF-A, VEGFR-1, and VEGFR-2 protein expression than bevacizumab monotherapy (*P* < 0.001). As shown in Figure 8B, the model group exhibited high expression of Notch1, Notch4, and DLL4, and all treatment groups produced a significant reduction in the protein levels of these molecules (*P* < 0.001). Compared with bevacizumab monotherapy, the combination of akiferidin with bevacizumab produced a further, significantly greater downregulation of Notch1, Notch4, and DLL4 protein expression (*P* < 0.0001). Together, these results indicate that akiferidin effectively inhibits the angiogenic factor VEGF-A and its receptors VEGFR-1 and VEGFR-2 in the cervical cancer model, and that this inhibition is markedly enhanced when akiferidin is combined with bevacizumab. Akiferidin also significantly downregulates Notch1, Notch4, and DLL4, key components of the DLL4/Notch signaling axis. These findings suggest that akiferidin suppresses tumor angiogenesis by coordinately modulating the VEGF and DLL4/Notch pathways, which may constitute a critical molecular mechanism for its inhibition of cervical cancer growth.

### 3.8 Molecular docking and molecular dynamics simulation

**FIGURE 9:**
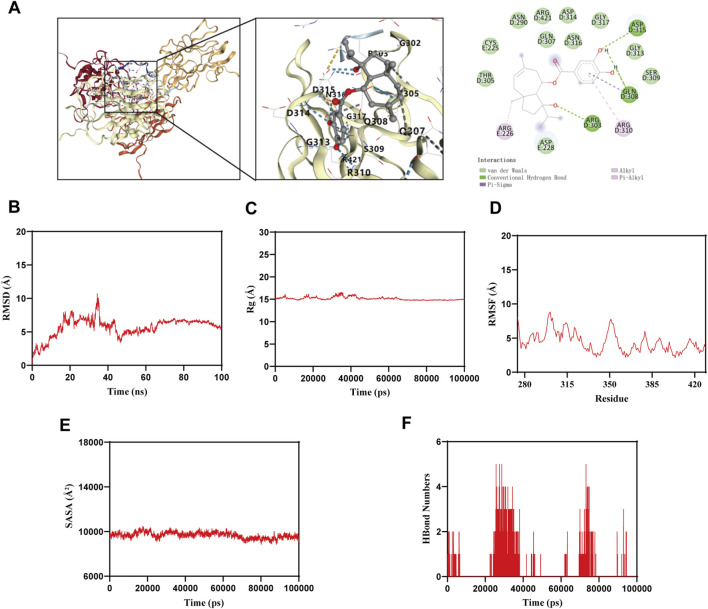
Molecular docking and molecular dynamics simulation of akiferidin with VEGF-A. **(A)** Molecular docking diagram of akiferidin with VEGF-A. **(B)** Root-mean-square deviation (RMSD) curve of the complex. **(C)** Radius of gyration (Rg) curve. **(D)** Root-mean-square fluctuation (RMSF) curve of residues. **(E)** Solvent-accessible surface area (SASA) curve. **(F)** Dynamic changes in the number of hydrogen bonds.

The results of Western blotting showed that the high-dose group of akiferidin significantly inhibited VEGF-A compared with the model group (Figure 9), suggesting that it may directly target VEGF-A to block the initiation of the VEGF signaling pathway. Therefore, molecular docking was performed using AutoDock Vina, and molecular dynamics simulations were carried out for 100 ns using GROMACS to elucidate the structural mechanisms of their interactions (Figure 9A). Akiferidin has five binding sites with VEGF-A, namely, arginine at positions 226, 303, and 310 of VEGF-A (ARG - 226, ARG - 303, ARG - 310), glutamic acid at position 308 (GLN - 308), and aspartic acid at position 315 (ASP - 315). Akiferidin forms various interactions with VEGF-A, including hydrogen bonds, π-alkyl interactions, and π-sigma interactions.

100 ns molecular dynamics simulation showed that the root-mean-square deviation (RMSD) of the complex stabilized at 7-8 Å after 60 ns (Figure 9B), indicating that the conformation of the system reached a kinetic equilibrium. The radius of gyration (Rg) of VEGF-A bound to akiferidin was maintained at 15-16 Å (Figure 9C). The system showed slight fluctuations during the motion and gradually stabilized, indicating that no significant conformational changes occurred in the complex during the motion. The root-mean-square fluctuation (RMSF) analysis revealed that the RMSF values of the complex were relatively low (mostly below 7 Å) (Figure 9D), indicating low flexibility and high stability. The solvent-accessible surface area (SASA) was stable at 10,000-11000 Å^2^ (Figure 9E). The complex system showed slight fluctuations, proving that the binding of small molecules affects the binding microenvironment and leads to a certain degree of SASA changes. The number of hydrogen bonds was dynamically maintained at 2-5 (Figure 9F), indicating good hydrogen-bond interactions between akiferidin and VEGF-A.

## 4 Discussion

Cervical cancer is one of the most common malignant tumors of the female reproductive system worldwide and seriously threatens the health of women. Its invasion and metastasis are the core reasons for poor patient prognosis (Doghish et al., 2023). Tumor angiogenesis, as a key driving force for tumor progression and metastasis, provides nutritional support and metabolic pathways for tumor cells and is a core link in the malignant progression of tumors (Gui et al., 2024). This process is precisely regulated by multiple signaling pathways such as VEGF and Notch, forming a complex cross-linked biomolecular network (Vimalraj, 2022; Zhang et al., 2025). Current clinical anti-angiogenic therapies mainly target a single pathway (such as VEGF pathway inhibitors like bevacizumab), but are often limited in efficacy and prone to drug resistance due to the activation of compensatory signaling pathways by tumor cells (such as abnormal upregulation of the Notch pathway), which greatly restricts the clinical therapeutic effect (Nittayaboon et al., 2025; Du et al., 2025). Therefore, developing botanical drugs that can simultaneously target multiple key regulatory nodes to break the compensatory activation cycle between pathways has become an important research direction to improve the effectiveness of anti-angiogenic treatment strategies. This study first systematically reported that akiferidin, a natural active plant metabolite from the Xinjiang medicinal plant *F*. *songorica*, significantly inhibits tumor angiogenesis and tumor growth in cervical cancer *in vitro* and *in vivo* models by inhibiting key molecules of the VEGF and DLL4-Notch signaling pathways. It also confirmed that akiferidin has a favorable combination effect with the clinical first-line anti-angiogenic drug bevacizumab and showed good *in vivo* safety. The results of this study provide new ideas, experimental evidence, and candidate molecules for the development of anti-cervical cancer drugs based on multi-target natural products.

This study systematically evaluated the *in vivo* antitumor activity and safety of akiferidin using a human cervical cancer U14-xenograft mouse model. The results showed that akiferidin significantly inhibited the growth of cervical cancer xenografts in a dose-dependent manner, as evidenced by the continuous reduction in tumor volume and significant decrease in tumor weight. The tumor-inhibitory effect of the akiferidin high-dose group was comparable to that of the clinical first-line chemotherapeutic drug paclitaxel. Moreover, no obvious systemic toxic reactions (such as diarrhea, and lethargy) were observed during the entire treatment period, which is highly consistent with the low-toxicity antitumor properties of Ferula plant extracts that have been reported (Malakotikhah et al., 2025; Aghaali et al., 2025). The low-toxicity advantage of natural products is an important prerequisite for their clinical translation. To further verify the safety of akiferidin, this study detected the body weight changes and core serum liver and kidney function indicators of tumor-bearing mice. The results showed that there was no significant difference in the body weight growth trend between the akiferidin-treated groups and the model group. The key liver and kidney function indicators in the serum were within the normal physiological range without any abnormal increase or decrease, indicating that akiferidin caused no significant damage to important organs such as the liver and kidneys of mice. Notably, the tumor-bearing mice in the model group had abnormally increased weights of the spleen, thymus, and kidneys due to tumor-related inflammation and immune disorders. However, akiferidin at each dose significantly reduced the abnormal weights of these organs, suggesting that it may exert a potential immunomodulatory effect by regulating the functions of immune cells in the tumor microenvironment. This finding further expands the application scenarios of its pharmacological value. In contrast, traditional chemotherapeutic drugs such as paclitaxel are often associated with serious adverse reactions, including myelosuppression, neurotoxicity, and liver and kidney damage, which greatly limit their clinical dosing and treatment duration (Huang et al., 2025; Wang S. et al., 2025; Prša et al., 2020). In summary, akiferidin exhibits strong antitumor activity while having good *in vivo* safety and dual-pathway targeting advantages. Moreover, it has a favorable combination effect when used in combination with bevacizumab. These core features make it a highly promising antitumor candidate plant metabolite, especially suitable for combination with other treatment modalities such as chemotherapy and immunotherapy to reduce the toxic and side effects of traditional treatments and overcome drug resistance, providing new strategies and experimental evidence for the combined treatment of cervical cancer.

The VEGF and Notch pathways have a close cross-regulatory network in the process of tumor angiogenesis, jointly maintaining the dynamic balance of angiogenesis. Their abnormal activation is a key driving factor for the disordered structure of tumor vessels, increased permeability, and enhanced invasiveness. The specific regulatory mechanisms are as follows: VEGF, as a core pro-angiogenic factor, mainly binds to VEGFR-2 on the surface of endothelial cells to activate downstream signaling pathways such as PI3K/Akt and MAPK, directly promoting endothelial cell proliferation, migration, and lumen formation. Meanwhile, the binding of VEGF to VEGFR-1 on endothelial cells can recruit bone marrow-derived endothelial progenitor cells and macrophages to the tumor microenvironment, working together with the VEGFR-2 signal to maintain the functional homeostasis of tumor vessels (Krishnan et al., 2025; Banerjee et al., 2024). The Notch signaling pathway regulates the fine-tuning process of angiogenesis in a contact-dependent manner between cells. The endothelial cell-specific ligands DLL4 and Jagged1 can bind to the Notch1/4 receptors on adjacent endothelial cells and activate the pathway. By regulating the expression of downstream target genes such as Hes1 and Hey1, they determine the fate differentiation of endothelial cells (such as the balance between tip cells and stalk cells), thereby participating in the shaping and maturation of the tumor vascular network (Negri et al., 2023; Vilchez Mercedes et al., 2022; Dong et al., 2020). The cross-talk between the two pathways is mainly realized through the “VEGF-DLL4-Notch negative feedback loop”: The combination of VEGF and VEGFR-2 can significantly induce the upregulation of DLL4 expression in endothelial cells. The binding of DLL4 to the Notch1/4 receptors of adjacent cells, through a negative feedback regulatory mechanism, inhibits the expression and activation of VEGFR-2, thereby inhibiting the excessive proliferation, migration of endothelial cells, and abnormal vascular sprouting, and maintaining the dynamic balance of angiogenesis (Li et al., 2021). When this regulatory loop is abnormally activated, it leads to increased tumor vascular density, structural disorder, decreased perfusion efficiency, and promotes the invasion and metastasis of tumor cells (Naseri et al., 2021).

To clarify the anti-angiogenic mechanism of akiferidin, this study detected the proportion of CD31-positive endothelial cells in tumor tissues by immunohistochemistry. The results showed that the proportion of CD31-positive vascular endothelial cells in tumor tissues was significantly reduced in each dose group of akiferidin, directly confirming its strong anti-angiogenic activity. Further detection by Western blot and immunohistochemistry found that akiferidin significantly downregulated the protein expression levels of key angiogenic factors in the VEGF pathway, such as VEGF-A, VEGFR-1, and VEGFR-2, in tumor tissues, providing direct molecular evidence for its anti-angiogenic effect. This is consistent with the previous finding from our research group that the ethanol extract of *F. songorica* can inhibit angiogenesis in zebrafish embryos, suggesting that akiferidin may be one of the core active plant metabolites of *F. songorica* for its anti-angiogenic effect. More importantly, the combination experiment confirmed that akiferidin has a significant combination antitumor effect when used in combination with bevacizumab. The degree of tumor regression and the reduction in vascular density were significantly better than those in the monotherapy groups of the two drugs. Mechanistic studies have shown that akiferidin can simultaneously inhibit the key molecules of the VEGF pathway (VEGF-A, VEGFR-1, VEGFR-2) and the Notch pathway (DLL4, Notch1, Notch4). This dual-inhibition effect can effectively disrupt the cancer-promoting signal amplification loop between the “VEGF-DLL4-Notch” pathways and avoid compensatory activation caused by single-pathway inhibition, which is the core molecular mechanism for its strong antitumor activity.

This exploratory study has several limitations. First, as a single-dose *in vivo* experiment, we did not perform a multi-dose gradient design or a formal pharmacodynamic interaction analysis to confirm a synergistic interaction between akiferidin and bevacizumab. Therefore, we report the enhanced antitumor efficacy of the combination as a combination effect rather than a confirmed synergistic effect; confirmation will require follow-up studies. Second, we did not characterize the pharmacokinetic profile of akiferidin, including its *in vivo* bioavailability and half-life in mice. Although the 20 mg/kg akiferidin dose and the 15 mg/kg paclitaxel positive-control dose were chosen based on pre-experimental validation and standard regimens for the U14 cervical cancer syngeneic model, formal pharmacokinetic studies are needed to optimize dosing and to define the exposure–efficacy relationship of akiferidin. Third, the preclinical model used here has inherent limitations for clinical translation. The mouse-derived U14 syngeneic model cannot fully reproduce the genomic and pathological features of human primary cervical cancer, nor the complex interactions between tumor cells and the human immune microenvironment, and results from the subcutaneous transplantation model have limited applicability to clinical settings.

In summary, this study elucidated the mechanism by which akiferidin, a novel multi-target natural plant metabolite, effectively inhibits cervical cancer angiogenesis and tumor growth by suppressing the VEGF and DLL4-Notch signaling pathways. Its favorable combination effect with bevacizumab and good safety profile make it a highly promising antitumor candidate plant metabolite. This study not only deepened the scientific understanding of the pharmacological material basis of Ferula plants but also provided important preclinical evidence for overcoming the limitations of current anti-angiogenic therapies and developing modern antitumor strategies derived from traditional botanical drugs. Future research will further conduct pharmacokinetic studies on akiferidin to clarify its *in vivo* metabolic characteristics, perform multi-dose gradient combination experiments to verify potential synergistic interactions, and explore combination therapy regimens with chemotherapeutic drugs and immune checkpoint inhibitors to provide more comprehensive experimental support for its clinical translation.

## Data Availability

The original contributions presented in the study are included in the article/supplementary material, further inquiries can be directed to the corresponding authors.

## References

[B1] Aghaali Z. Zargar M. Naghavi M. R. (2025). Encapsulation of Ferula-derived bioactive compounds in nanoparticles: a promising therapeutic route for cancers and infectious diseases. Int. Immunopharmacol. 157, 114705. 10.1016/j.intimp.2025.114705 40306115

[B2] Banerjee N. Roy L. Panda S. Roychowdhury T. Chatterjee S. (2024). In silico-designed G-quadruplex targeting peptide attenuates VEGF-A expression, preventing angiogenesis in cancer cells. Chem. Biol. Drug Des. 104 (6), e70018. 10.1111/cbdd.70018 39704035

[B3] Burley S. K. Bhikadiya C. Bi C. Bittrich S. Chao H. Chen L. (2023). RCSB protein data bank (RCSB.org): delivery of experimentally-determined PDB structures alongside one million computed structure models of proteins from artificial intelligence/machine learning. Nucleic Acids Res. 51 (D1), D488–D508. 10.1093/nar/gkac1077 36420884 PMC9825554

[B4] Doghish A. S. Ali M. A. Elyan S. S. Elrebehy M. A. Mohamed H. H. Mansour R. M. (2023). miRNAs role in cervical cancer pathogenesis and targeted therapy: signaling pathways interplay. Pathol. Res. Pract. 244, 154386. 10.1016/j.prp.2023.154386 36868096

[B5] Dong J. Yang W. Han J. Cheng R. Li L. (2020). Effects of notch signaling components from breast cancer cells treated in culture with resveratrol. Res. Vet. Sci. 132, 369–378. 10.1016/j.rvsc.2020.07.017 32745729

[B6] Du P. Li Y. Han A. Wang M. Liu J. Piao Y. (2025). β-lapachone suppresses carcinogenesis of cervical cancer via interaction with AKT1. Front. Pharmacol. 16, 1509568. 10.3389/fphar.2025.1509568 40051559 PMC11882534

[B7] Gui Z. Ye Y. Li Y. Ren Z. Wei N. Liu L. (2024). Construction of a novel cancer-associated fibroblast-related signature to predict clinical outcome and immune response in cervical cancer. Transl. Oncol. 46, 102001. 10.1016/j.tranon.2024.102001 38850798 PMC11214323

[B8] Huang S. Huang Z. Sun Z. Xie T. Zhu X. (2025). Real-world performance of the machine learning-based prediction of chemotherapy-associated adverse effects in lung cancer. Oncol. Lett. 31 (1), 24. 10.3892/ol.2025.15377 41246558 PMC12612794

[B9] Kim S. Lee J. Jo S. Brooks C. L. Lee H. S. Im W. (2017). CHARMM-GUI ligand reader and modeler for CHARMM force field generation of small molecules. J. Comput. Chem. 38 (21), 1879–1886. 10.1002/jcc.24829 28497616 PMC5488718

[B10] Krishnan G. H. N N. K. Parthiban V. Sarkar S. K S. (2025). *In silico* identification of 9-amino camptothecin as a dual inhibitor of VEGF-A and VEGF-B in N1-N8 breast cancer. Biotechnol. Appl. Biochem. 10.1002/bab.70070 41147711

[B11] Li B. Tong T. Ren N. Rankin G. O. Rojanasakul Y. Tu Y. (2021). Theasaponin E1 inhibits platinum-resistant ovarian cancer cells through activating apoptosis and suppressing angiogenesis. Molecules 26 (6), 1681. 10.3390/molecules26061681 33802884 PMC8002815

[B12] Li Y. Song W. Gao P. Guan X. Wang B. Zhang L. (2025). Global, regional, and national burden of breast, cervical, uterine, and ovarian cancer and their risk factors among women from 1990 to 2021, and projections to 2050: findings from the global burden of disease study 2021. BMC Cancer 25 (1), 330–346. 10.1186/s12885-025-13741-9 39988683 PMC11849330

[B13] Li T. Zhang H. Lian M. He Q. Lv M. Zhai L. (2025). Global status and attributable risk factors of breast, cervical, ovarian, and uterine cancers from 1990 to 2021. J. Hematol. Oncol. 18 (1), 5. 10.1186/s13045-025-01660-y 39794860 PMC11721161

[B14] Limones-Gonzalez J. E. Aguilar Esquivel P. Vazquez-Santillan K. Castro-Oropeza R. Lizarraga F. Maldonado V. (2024). Changes in the molecular nodes of the notch and NRF2 pathways in cervical cancer tissues from the precursor stages to invasive carcinoma. Oncol. Lett. 28 (5), 522. 10.3892/ol.2024.14655 39268158 PMC11391250

[B15] Liu Q. Y. Zhang H. Y. Zhao S. J. (2023). Study on *in vivo* and *in vitro* inhibition of 5 species of smelly ferula from Xinjiang on U14 cell of mouse cervical cancer. J. Xinjiang Med. Univ. 46 (11), 1432–1437.

[B16] Ma Y. Xiu Z. Zhou Z. Huang B. Liu J. Wu X. (2019). Cytochalasin H inhibits angiogenesis via the suppression of HIF-1α protein accumulation and VEGF expression through PI3K/AKT/P70S6K and ERK1/2 signaling pathways in non-small cell lung cancer cells. J. Cancer 10 (9), 1997–2005. 10.7150/jca.29933 31205560 PMC6548170

[B17] Malakotikhah F. Shahanipour K. Monajemi R. Ahadi A. M. Rastegari A. A. (2025). Investigating the effect of ferula assafoetida L. extract and farnesiferol on adapter activation and inhibition of metastasis of genes involved in the severity of malignancy in breast cancer cell lines. Food Sci. Nutr. 13 (8), e70686. 10.1002/fsn3.70686 41322087 PMC12658380

[B18] Naseri M. Saeednejad Zanjani L. Vafaei S. Gheytanchi E. Abolhasani M. Bozorgmehr M. (2021). Increased cytoplasmic expression of DLL4 is associated with favorable prognosis in colorectal cancer. Future Oncol. 17 (24), 3231–3242. 10.2217/fon-2020-0840 34156260

[B19] Negri F. Bottarelli L. Pedrazzi G. Maddalo M. Leo L. Milanese G. (2023). Notch-Jagged1 signaling and response to bevacizumab therapy in advanced colorectal cancer: a glance to radiomics or back to physiopathology? Front. Oncol. 13, 1132564. 10.3389/fonc.2023.1132564 36925919 PMC10011088

[B20] Nittayaboon K. Molika P. Bissanum R. Leetanaporn K. Chumsuwan N. Navakanitworakul R. (2025). Bone marrow mesenchymal stem cell-derived exosomes modulate chemoradiotherapy response in cervical cancer spheroids. Pharm. (Basel) 18 (7), 1050. 10.3390/ph18071050 40732337 PMC12299787

[B21] Prša P. Karademir B. Biçim G. Mahmoud H. Dahan I. Yalçın A. S. (2020). The potential use of natural products to negate hepatic, renal and neuronal toxicity induced by cancer therapeutics. Biochem. Pharmacol. 173, 113551. 10.1016/j.bcp.2019.06.007 31185225

[B22] Saleh A. Mansour D. F. Raslan N. A. Galal O. Ibrahim S. Hafez M. M. (2025). Targeting inflammation-driven tumor progression with zafirlukast via modulation of Jagged-1/Notch-1/Hes-1, Wnt-4/β-catenin, and VEGF signaling pathways in mice. Naunyn Schmiedeb. Arch. Pharmacol. 398 (12), 17609–17632. 10.1007/s00210-025-04344-z 40522499

[B23] Sun H. Wang G. Ren C. Zhang X. Zhao P. Guo B. (2025). Erianin inhibits cell migration and induces apoptosis by inhibiting VEGF-α/PI3K/AKT signaling pathway in malignant melanoma. Sci. Rep. 15 (1), 15766. 10.1038/s41598-025-99383-0 40328945 PMC12056193

[B24] Valdés-Tresanco M. S. Valdés-Tresanco M. E. Valiente P. A. Moreno E. (2021). gmx_MMPBSA: a new tool to perform end-state free energy calculations with GROMACS. J. Chem. Theory Comput. 17 (10), 6281–6291. 10.1021/acs.jctc.1c00645 34586825

[B25] Vilchez Mercedes S. A. Bocci F. Ahmed M. Eder I. Zhu N. Levine H. (2022). Nrf2 modulates the hybrid epithelial/mesenchymal phenotype and notch signaling during collective cancer migration. Front. Mol. Biosci. 9, 807324. 10.3389/fmolb.2022.807324 35480877 PMC9037689

[B26] Vimalraj S. (2022). A concise review of VEGF, PDGF, FGF, notch, angiopoietin, and HGF signalling in tumor angiogenesis with a focus on alternative approaches and future directions. Int. J. Biol. Macromol. 221, 1428–1438. 10.1016/j.ijbiomac.2022.09.129 36122781

[B27] Wang D. Liu X. Hong W. Xiao T. Xu Y. Fang X. (2024). Muscone abrogates breast cancer progression through tumor angiogenic suppression via VEGF/PI3K/Akt/MAPK signaling pathways. Cancer Cell Int. 24 (1), 214. 10.1186/s12935-024-03401-6 38898449 PMC11188526

[B28] Wang J. Zhang J. Wang T. Rong C. (2025). Notch signaling pathway in cervical cancer: from molecular mechanism to therapeutic potential. Cell. Signal. 135, 112042. 10.1016/j.cellsig.2025.112042 40752538

[B29] Wang S. She C. Xu G. Hu X. Yan W. (2025). A study on the improvement of quality of survival in cervical cancer patients after chemoradiotherapy through an integrated traditional Chinese and Western medicine therapy based on a syndrome differentiation-driven dynamic intervention strategy. Oncology. 10.1159/000548661 41343416

[B30] Wariss H. M. Yang L. Ahmad S. Yaseen A. Riaz M. Tariq A. (2026). The ethnomedicinal, pharmacological, and phytochemical potential of Ferula sinkiangensis K.M.Shen: an updated and comprehensive review. J. Ethnopharmacol. 354, 120444. 10.1016/j.jep.2025.120444 40854415

[B31] Yilimire A. Zhang H. Y. Zhao S. J. (2023). Anti-gastric cancer effect of alcohol extracts from five kinds of fragrant ferula in Xinjiang. China Pharm. 32 (08), 29–34.

[B32] Zhang L. Xu Q. Sun G. Zhang X. Xue J. Yao C. (2025). Farrerol inhibits proliferation and migration of colorectal cancer via the VEGF signaling pathway: evidence from network pharmacology, molecular docking, molecular dynamics simulation, and *in vitro* experiments. Front. Pharmacol. 16, 1717293. 10.3389/fphar.2025.1717293 41415563 PMC12708557

[B33] Zhou Y. Zhang Y. Bao J. Chen J. Song W. (2022). Low temperature plasma suppresses lung cancer cells growth via VEGF/VEGFR2/RAS/ERK axis. Molecules 27 (18), 5934. 10.3390/molecules27185934 36144670 PMC9502791

[B34] Zhou L. Li Y. Wang H. Qin R. Han Z. Li R. (2025). Global cervical cancer elimination: quantifying the status, progress, and gaps. BMC Med. 23 (1), 67–73. 10.1186/s12916-025-03897-3 39901174 PMC11792702

